# Supplementary material for: Predicting wind-driven spatial deposition through simulated color images using deep autoencoders
Source: Sci Rep. 2023 Jan 25;13:1394. doi: 10.1038/s41598-023-28590-4 (PMC9876895; doi:10.1038/s41598-023-28590-4)
Supplement: Supplementary file 1 — Supplementary Information. [file 41598_2023_28590_MOESM1_ESM.pdf]

Supplementary material

Appendix A : Model interpretability

Figure A1 shows the mean Pearson correlation between the four inputs of the bottleneck model and each of the 25×25 pixels of the latent space. The figure was generated using the training data (10,530 images). The test data was not used for this calculation because we are interpreting the trained model and not evaluating its performance in this case. As expected for the variable s_x, the correlation changes substantially along the horizontal axis, and there is roughly no change along the vertical axis. Conversely, for s_y, the correlation variation is substantial through the vertical axis while almost nonexistent through the horizontal axis. We have the same behavior for w_u (the horizontal component of the wind velocity) and w_v (the vertical component of the wind velocity).

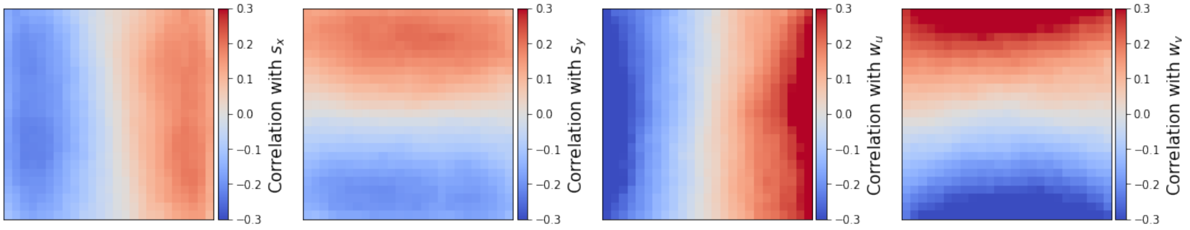

Figure A1. Correlation between the two-dimensional latent space of shape $[25, 25, 1]$ and the inputs of interest, source location (s_x, s_y) and wind velocity (w_u, w_v), on the training data (10,530 images).
